# Non-typhoidal *Salmonella* bloodstream infections in Kisantu, DR Congo: Emergence of O5-negative *Salmonella* Typhimurium and extensive drug resistance

**DOI:** 10.1371/journal.pntd.0008121

**Published:** 2020-04-02

**Authors:** Bieke Tack, Marie-France Phoba, Barbara Barbé, Lisette M. Kalonji, Liselotte Hardy, Sandra Van Puyvelde, Brecht Ingelbeen, Dadi Falay, Dauly Ngonda, Marianne A. B. van der Sande, Stijn Deborggraeve, Jan Jacobs, Octavie Lunguya

**Affiliations:** 1 Department of Clinical Sciences, Institute of Tropical Medicine, Antwerp, Belgium; 2 Department of Microbiology and Immunology, KU Leuven, Belgium; 3 Department of Microbiology, National Institute for Biomedical Research, Kinshasa, Democratic Republic of the Congo; 4 Department of Microbiology, University Teaching Hospital of Kinshasa, Kinshasa, Democratic Republic of the Congo; 5 Department of Biomedical Sciences, Institute of Tropical Medicine, Antwerp, Belgium; 6 Laboratory of Medical Microbiology, Vaccine & Infectious Disease Institute, Universiteit Antwerpen, Antwerp, Belgium; 7 Wellcome Trust Sanger Institute, Hinxton, United Kingdom; 8 Department of Public Health, Institute of Tropical Medicine, Antwerp, Belgium; 9 Department of Pediatrics, University Hospital of Kisangani, Kisangani, Democratic Republic of the Congo; 10 Global Health Centre, Julius Center for Health Sciences and Primary Care, University Medical Centrum Utrecht, Utrecht University, Utrecht, Netherlands; International Vaccine Institute, REPUBLIC OF KOREA

## Abstract

**Background:**

Non-typhoidal *Salmonella* (NTS) are a major cause of bloodstream infection (BSI) in sub-Saharan Africa. This study aimed to assess its longitudinal evolution as cause of BSI, its serotype distribution and its antibiotic resistance pattern in Kisantu, DR Congo.

**Methods:**

As part of a national surveillance network, blood cultures were sampled in patients with suspected BSI admitted to Kisantu referral hospital from 2015–2017. Blood cultures were worked-up according to international standards. Results were compared to similar data from 2007 onwards.

**Results:**

In 2015–2017, NTS (n = 896) represented the primary cause of BSI. NTS were isolated from 7.6% of 11,764 suspected and 65.4% of 1371 confirmed BSI. In children <5 years, NTS accounted for 9.6% of suspected BSI. These data were in line with data from previous surveillance periods, except for the proportion of confirmed BSI, which was lower in previous surveillance periods. *Salmonella* Typhimurium accounted for 63.1% of NTS BSI and *Salmonella* Enteritidis for 36.4%. Of all *Salmonella* Typhimurium, 36.9% did not express the O5-antigen (*i*.*e*. variant Copenhagen). O5-negative *Salmonella* Typhimurium were rare before 2013, but increased gradually from then onwards. Multidrug resistance was observed in 87.4% of 864 NTS isolates, decreased ciprofloxacin susceptibility in 7.3%, ceftriaxone resistance in 15.7% and azithromycin resistance in 14.9%. A total of 14.2% of NTS isolates, that were all *Salmonella* Typhimurium, were multidrug resistant and ceftriaxone and azithromycin co-resistant. These *Salmonella* isolates were called extensively drug resistant. Compared to previous surveillance periods, proportions of NTS isolates with resistance to ceftriaxone and azithromycin and decreased ciprofloxacin susceptibility increased.

**Conclusion:**

As in previous surveillance periods, NTS ranked first as the cause of BSI in children. The emergence of O5-negative *Salmonella* Typhimurium needs to be considered in the light of vaccine development. The high proportions of antibiotic resistance are worrisome.

## Author summary

We reviewed bloodstream infections caused by non-typhoidal *Salmonella* over a 10 year period (2007–2017) in the Democratic Republic of the Congo, with Kisantu in the Kongo Central province as the major sampling site. Non-typhoidal *Salmonella* were the most common cause of bloodstream infections in children in a sentinel network in DR Congo. The disease mainly occurred in young children and had a high mortality. We showed that non-typhoidal *Salmonella* persisted to frequently cause bloodstream infections in children under 5 years old. *Salmonella* Enteritidis and *Salmonella* Typhimurium were the main serotypes causing non-typhoidal *Salmonella* bloodstream infections. The emergence of O5-antigen negative *Salmonella* Typhimurium (*i*.*e*. variant Copenhagen) was observed and this needs to be considered when developing vaccines. Multidrug resistance to ampicillin, trimethoprim-sulfamethoxazole and chloramphenicol was widespread during the whole decade. From 2013 onwards, resistance to all other available antibiotics, *i*.*e*. third generation cephalosporins, fluoroquinolones and azithromycin, emerged. Likewise, extensive drug resistance (multidrug, ceftriaxone and azithromycin co-resistance) and multidrug resistance combined with decreased ciprofloxacin susceptibility in *Salmonella* Typhimurium emerged. Unfortunately, there are no clinical studies on antibiotic treatment in non-typhoidal *Salmonella* bloodstream infection that could allow the development of evidence based treatment guidelines. Therefore, dedicated observational studies and clinical studies are urgently needed to optimize antibiotic stewardship.

## Introduction

Non-typhoidal *Salmonella* (NTS) is one of the predominant pathogens causing bloodstream infections (BSI) in sub-Saharan Africa [1,2]. In 2017, it was estimated that sub-Saharan Africa accounted for 421,600 of the 535,000 cases of invasive NTS infections in the world. The geographical distribution of NTS BSI is linked to the geographical distribution of its risk factors. NTS BSI mainly occurs in individuals with underlying medical conditions, *e*.*g*. HIV-infected individuals and (young) children with *Plasmodium falciparum* malaria infections, anemia and/or malnutrition. The underlying medical conditions and age of the affected individual influence the case-fatality rate of NTS BSI. In low sociodemographic developed areas in sub-Saharan Africa the case-fatality rate is estimated at 15.8% (range: 13–28%). In NTS BSI in sub-Saharan Africa, the serotypes that are most frequently observed are *Salmonella enterica* subspecies *enterica* (hereafter briefly called *Salmonella*) Typhimurium, Enteritidis and Dublin [1,3]. In these NTS, multidrug resistance (MDR) (co-resistance to ampicillin, trimethoprim-sulfamethoxazole and chloramphenicol) is widespread [1,4,5]. The alternative treatment options are confined to third generation cephalosporins (*e*.*g*. ceftriaxone), fluoroquinolones (*e*.*g*. ciprofloxacin) and azithromycin. To assist antibiotic stewardship, the essential medicines list from the World Health Organization (WHO) classified antibiotics in three categories: key access, watch and reserve group antibiotics. Third generation cephalosporins, fluoroquinolones and azithromycin are watch group antibiotics and thus prioritized as key targets of stewardship programs and monitoring [6].

In the Democratic Republic of the Congo (DRC), the burden of NTS BSI is high. In DRC, most NTS BSI cases are children. A study from Tshopo Province in DRC showed that most of children with NTS BSI were co-infected with *Plasmodium falciparum* malaria and many of them were anemic. The study reported a case fatality of 13.3%. Blood culture surveillance studies in DRC further showed that *Salmonella* Typhimurium and Enteritidis were the principal serotypes [7,8]. Most of the NTS isolates assessed were multidrug resistant, but also sporadic ceftriaxone and azithromycin resistance and decreased ciprofloxacin susceptibility were observed [7,8].

The present study reports on all NTS BSI between 2015 and 2017 that were observed at Kisantu general referral hospital. We assessed the number of NTS BSI as proportion of all suspected and confirmed BSI, the serotype distribution and antibiotic resistance profiles. Secondly, we compared current findings with previous surveillance periods, i.e. 2007–2010 and 2011–2014 [7]. Finally, where appropriate, data on NTS BSI in other blood culture surveillance sites in DRC are briefly mentioned.

## Methods

### Study setting and period

In 2007, the DRC National Institute for Biomedical Research (INRB, Kinshasa) set up a microbiological surveillance network in collaboration with the Institute of Tropical Medicine (ITM), Antwerp, Belgium. The network started in healthcare facilities in Kinshasa and later extended to 3 sentinel hospitals: Kisantu general referral hospital (Province Bas-Congo, presently named Kongo Central), Bwamanda general referral hospital (Province Equateur, presently named Sud-Ubangi) and Kisangani university referral hospital (Province Orientale, presently named Tshopo).

The present study focused on the data from blood culture isolates from Kisantu general referral hospital from the period January 2015 –October 2017. In addition, the results from the blood culture surveillance in other sites were briefly mentioned. Kisantu hospital is a 274-bed district hospital. The referral district has a surface of 2400 km^2^ and consists of 4 semi-urban health areas surrounding the hospital and 11 more remote rural health areas. While the district counted approximately 140,000 inhabitants in 2007, its population grew to approximately 190,000 inhabitants in 2017. From these inhabitants, approximately 45% lived in the hospital surrounding semi-urban areas; a proportion that remained stable over time. Each health area is served by a health center that manages and refers its patients to the hospital according to the national guidelines. In the hospital, referred patients benefit from a flat-fee system that was installed in 2009. As a result of the flat-fee system, the proportion of referred hospital-admitted patients increased from 6.1% in 2008 to 93.9% in 2011. The hospital bed occupancy rate increased from 52% in 2008 to over 85% in 2011 [9]. The bed occupancy rate reached 144% in 2017 in the pediatric ward, which accounted for 52% of all hospital admissions in 2017. Among all hospital wards, integration of blood culture sampling in routine 24/7 clinical practice was best in the pediatric ward.

*P*. *falciparum* malaria is hyperendemic in DRC [10]. According to a national health survey in 2013–2014, 24% of children in Bas-Congo between 6–59 months had a positive blood microscopy test. According to the same survey, 46% of children under 5 years old were chronically malnourished and 11% of them were acutely malnourished. The survey reported an HIV-prevalence of 0.2% in adults aged 15–49 years [11]. The extended national immunization program in DRC includes Bacille Calmette-Guérin, polio, diphtheria, pertussis, tetanus, hepatitis B, measles, *Haemophilus influenzae*, yellow fever and the 13-valent pneumococcal vaccine [12]. In 2013–2014, 55% of all children aged 12–23 month in Bas-Congo had received all recommended vaccines [11]. A detailed overview of the country characteristics of DRC can be found in S1 Table.

### Blood culture indications, sampling and culturing

Indications for blood culture sampling were an axillary body temperature of ≥ 38.0°C, ≤ 36°C or history of fever during the last 48 hours with signs of severity such as hypotension, confusion and increased respiratory rate, with suspicion of severe localized infections (pneumonia, meningitis, complicated urinary tract infection, osteomyelitis and arthritis, severe skin and soft tissue infections, gynecological and abdominal infections/peritonitis) or with suspicion of other severe infections: sepsis, typhoid fever, and severe malaria [13]. Additional criteria for blood culture sampling in the neonatal period were premature rupture of membranes, intrapartum fever, low Apgar score, or specific symptoms such as tachypnea, cyanosis, and lethargy. Basic demographic (age, gender, residence) and clinical data (presumptive clinical diagnosis/focus of infection, prehospital antibiotic and antimalarial treatment) as documented on the lab request form were registered in an Excel database (Microsoft Corporation, Redmond, Washington, USA).

In adults (>14 years old), 2 x 10 ml of blood from separate venipunctures was sampled and inoculated in aerobic BacT/ALERT bottles (bioMérieux, Marcy-L’Etoile, France). In children (≤ 14 years old): 1 x 4 ml of blood was sampled in pediatric BacT/ALERT bottles. Blood cultures were collected and worked-up free of charge and processed on site in Kisantu hospital and Kisangani university referral hospital to allow integration in routine clinical care. In Bwamanda, incubated blood cultures were shipped to INRB for further work-up. Blood cultures sampling could be performed at every day of the week and every hour of the day. Blood culture work-up was done on a daily basis, including in the weekend. Blood cultures were incubated at 35°C with a short needle-to-incubator transport time for 7 consecutive days with daily visual check of the colorimetric growth indicator. When growth was detected, Gram stain with subculture on appropriate culture media and phenotypic identification by standard biochemical methods [8] was performed. As part of patient care and laboratory surveillance, antibiotic susceptibility testing with the disk diffusion method (Neo-Sensitabs, Rosco, Taastrup, Denmark) was performed on site according to the consecutive CLSI guidelines along the study period [14]. Isolates were stored on Tryptone Soya Agar (Oxoid) and shipped to INRB and ITM for in-depth reference testing.

### Reference testing: Serotyping and antibiotic susceptibility testing

At INRB and ITM, identification and antibiotic susceptibility testing of pathogens were repeated in batch for all isolates. Identification was done using standard biochemical methods [8] and serotyping was done according to the Kaufman-White scheme [15] using commercial antisera (Pro-lab diagnostics, Toronto, Canada and Sifin, Berlin, Germany). NTS isolates with inconclusive serotype or with a serotype other than Typhimurium and Enteritidis, were submitted to the Belgian reference laboratory for *Salmonella* and *Shigella* serotyping (Sciensano, Brussels). For quality control, a random selection of 10% of all NTS isolates was submitted to the same reference laboratory. This quality control step revealed the presence of O5-negative *Salmonella* Typhimurium. O5-antigen testing was included in the routine serotyping scheme at ITM since 2014.

Antimicrobial susceptibility testing was done following CLSI guidelines using disk diffusion (Neo-Sensitabs, Rosco, Taastrup, Denmark) and by assessing Minimum Inhibitory Concentrations (MIC) with the E-test macromethod (bioMérieux, Oxoid) for ciprofloxacin and azithromycin. In addition, surrogate disk diffusion testing with nalidixic acid and pefloxacin was performed to predict decreased susceptibility or resistance to ciprofloxacin. Quality control was performed using American Type Culture Collection (ATCC) *Escherichia coli* 25922, *Staphylococcus aureus* 29213, and *Klebsiella pneumoniae* 700603. The results were interpreted according to CLSI M100-29 and intermediate-susceptible isolates were grouped together with the resistant ones [14,16]. Since 2016, CLSI no longer recommends the use of nalidixic acid, so diameters were interpreted according to CLSI M100-26 [17]. NTS was considered as resistant to azithromycin at MIC-values > 16 mg/L, in analogy with the epidemiological cut-off value for *Salmonella* Typhi [14,18] and epidemiological data of MIC-values of intestinal NTS [19–22].

### Definitions and terminology

Only isolates that were available for reference testing at INRB/ITM were reported. For surveillance purposes, only the first isolate per bloodstream infection episode was considered for analysis. A confirmed BSI episode, briefly referred to as confirmed BSI, was defined per patient as (i) the initial isolation of a pathogen from a blood culture, (ii) the subsequent isolation of a pathogen different from the initial pathogen (species-level) from a subsequent blood culture or (iii) the isolation of the same pathogen (species-level) after at least a 14-day interval after isolation of the initial pathogen from a subsequent blood culture. The latter was called a recurrent BSI. In analogy to the term confirmed BSI, the term “suspected BSI” was used to indicate all blood cultures in a single patient sampled during a 14-day period starting at the moment of blood culture sampling [16,23,24]. Coagulase-negative *Staphylococcus*, *Bacillus* species, *Corynebacterium* species, *Micrococcus* species, *Cutibacterium* (previously *Propionibacterium*) species and *Lactobacillus* species were considered as environmental or skin contaminants. Viridans streptococci were considered as contaminants if they were isolated only once per BSI episode, but as pathogens if they were isolated in more than one blood culture from the same BSI episode. All other bacteria were classified as pathogens [25].

*Salmonella* Typhimurium that do not react with O5-antiserum, also known as *Salmonella* Typhimurium variant Copenhagen [15], are referred to as O5-negative *Salmonella* Typhimurium. “Regular” *Salmonella* Typhimurium, *i*.*e*. expressing the O5-epitope, are referred to as O5-positive *Salmonella* Typhimurium.

The term “decreased ciprofloxacin susceptibility” (DCS) was used to indicate *Salmonella* isolates with a ciprofloxacin MIC-value > 0.064 mg/L and < 1 mg/L [4,14]. Table 1 gives an overview of the definitions of multidrug resistance (MDR), extensive drug resistance (XDR) and pandrug resistance (PDR) in *Salmonella* used in this report[26]. Some authors suggest to interpret pandrug resistance as resistance to all antibiotic classes available for empirical treatment [27]. In many low and middle income countries, including in DRC, carbapenems are not available or affordable. As such, carbapenem susceptibility was not taken into account in this report and PDR was defined as combined MDR + DCS + ceftriaxone and azithromycin resistance.

**Table 1:** Definitions of multidrug resistance (MDR), extensive drug resistance (XDR) and pandrug resistance (PDR) in *Salmonella*.

	MDR	XDR			PDR
Ampicillin	R	R	R	R	R
Trimethoprim-sulfamethoxazole	R	R	R	R	R
Chloramphenicol	R	R	R	R	R
Third generation cephalosporins	S	R	R	S	R
DCS or fluoroquinolone resistance	S	S	R	R	R
Azithromycin	S	R	S	R	R
*Meropenem*	*S*	*S*	*S*	*S*	*S*

XDR was formulated in analogy with previous publications on *Salmonella* [26–28]. Because PDR was interpreted as resistance to all antibiotic classes available for empirical treatment in low and middle income countries [27], carbapenem susceptibility was not taken into account. Abbreviations: DCS: decreased ciprofloxacin susceptibility

### Comparison with previous surveillance periods

Databases from previous surveillance periods (April 2007 –January 2011 (further referred to as “2007–2010” [8]) and January 2011 –December 2014 (further referred to as “2011–2014” [7]) were compiled with the database from 2015–2017 to create a comprehensive database of the 10 years of blood culture surveillance in DRC. In 2015, the 11 former DRC-provinces were split in the current 26 provinces. To facilitate comparison with the previous reported surveillance reports, the former province names are used in this publication. If it had not been performed yet, O5 serotyping of *Salmonella* Typhimurium from previous surveillance periods (stored at -80°C) was performed. From 2007–2010, antibiotic susceptibility testing was partially done using the Vitek II system (Card AB AST-N156, bioMérieux), as described in Lunguya et al. 2013 [8]. Interpretation of antibiotic susceptibility testing was harmonized according to the breakpoints in CLSI M100-29, which was valid at the moment of analysis [14].

### Statistical analysis

All data were compiled in an Excel database (Microsoft Corporation, Redmond, Washington, USA); Stata software, version 15 (Stata Corp, College Station, Texas) was used for statistical analysis. The number of suspected and confirmed BSI was calculated, by underlying pathogen, by province and by surveillance period. Where appropriate, a stratified analysis per age category was done. The following age categories were used: children < 5 years, children ≥ 5 years old and adults. Antibiotic resistance was expressed as the proportion of NTS isolates resistant to the tested antibiotic or antibiotic combination (MDR, XDR & PDR: Table 1) from all the (single) NTS isolates for which susceptibility data were available for all antibiotics tested at reference level. The χ2 test was used to assess whether observed differences in proportions were statistically significant (p-value <0.05). Age distributions were compared using the Wilcoxon-Mann-Whitney nonparametric test. Simple linear regression was used to assess linear trends in proportions over the years.

### Ethics statement

The present study complies with the WHO [29] and international (Council for International Organizations of Medical Sciences [30] and European Centre for Disease Prevention and Control [31]) guidelines on antibiotic surveillance and antibiotic stewardship for which no recommendation for an informed consent has been issued. Ethical approval for the microbiological surveillance study was granted by the Institutional Review Board of ITM (ref. 613/08), the Ethics Committee of Antwerp University (ref. 8/20/96), and the School of Public Health (Ecole de santé public) of Kinshasa in DRC (ref. 074/2017).

## Results

### Overview of sampling from 2015–2017: Most BSI were sampled in children

**Fig 1:**
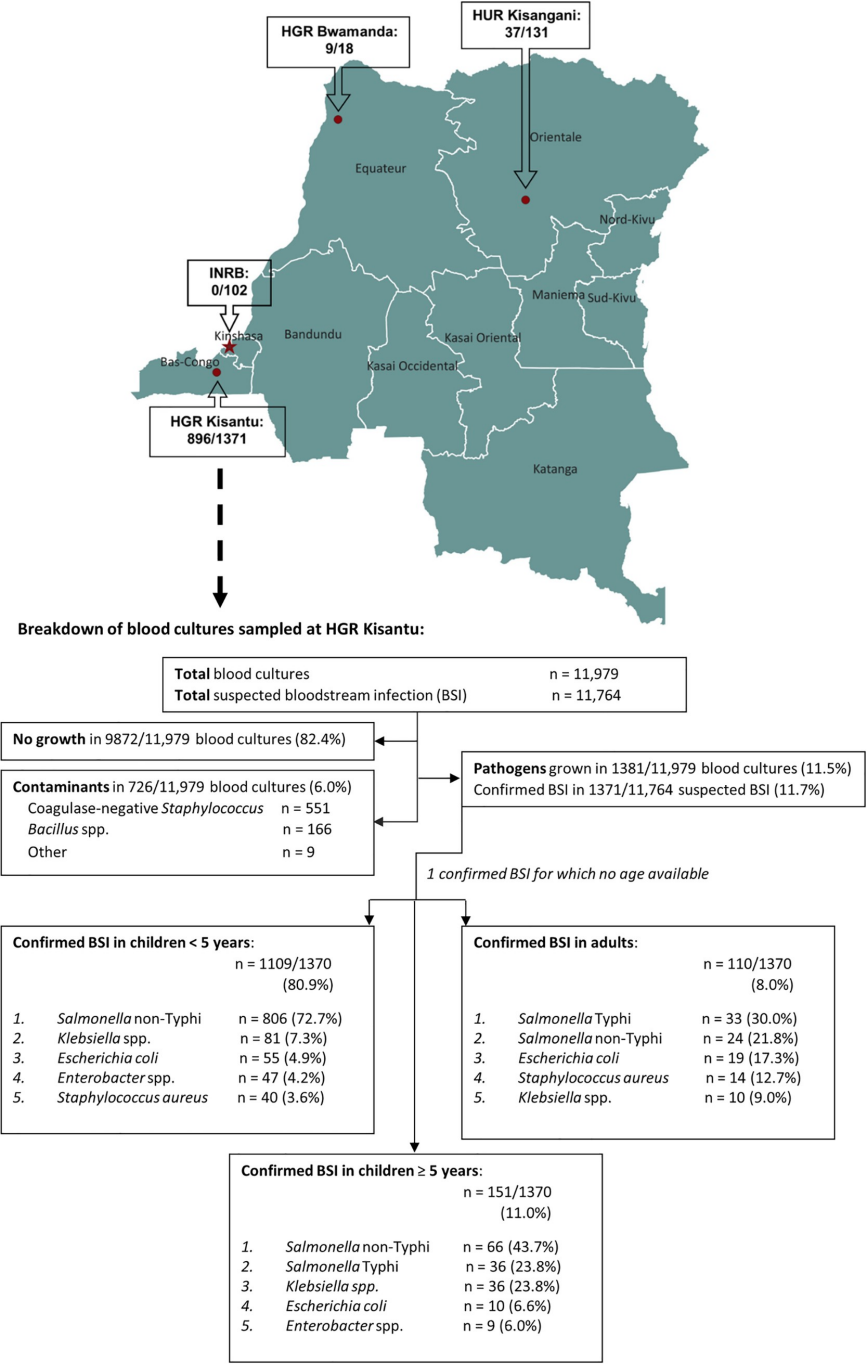
Overview of NTS BSI per surveillance site and breakdown of blood cultures sampled at Kisantu general referral hospital from 2015 till 2017. The number of bloodstream infections caused by NTS are presented as part of the number of all confirmed bloodstream infections per surveillance site. Unless otherwise stated, data are presented as first isolates per bloodstream infection episode. In the top 5 of pathogens, the percentages represent the proportion of all pathogens in children <5 years, ≥ 5 years or adults for whom information about age was available (n = 1109, 151 and 110, respectively). The map was developed with QGIS (version 3.4.3), the spatial dataset for the country and province boundaries can be accessed at https://data.humdata.org/dataset/drc-administrative-boundaries-levels-0-2 (date of access 08/10/2018), the location of the surveillance sites was based on open street map data integrated in the QGIS software. Abbreviations: HGR: general referral hospital, HUR: university referral hospital, INRB: National Institute for Biomedical Research, BSI: bloodstream infection.

From 2015 till 2017, 11,979 blood cultures, accounting for 11,764 suspected BSI, were sampled at Kisantu hospital. Blood culture contaminants were found in 6.0%. Pathogens were isolated from 1370 suspected BSI, i.e. a positivity rate of 11.7% (Fig 1). Children accounted for 10,739 of 11,737 (91.4%) suspected BSI for which data on age were available. From these suspected BSI in children, 8394 (78.2%) were sampled from children < 5 years old. The positivity rate did not significantly differ between children and adults (11.7% from 10,739 suspected BSI in children vs. 11.0% from 998 suspected BSI in adults, p = 0.5). However, the positivity rate in children ≥ 5 years old was significantly lower than in children < 5 years (6.4% vs 13.2% with p<0.001, respectively).

### Proportion of bloodstream infections: NTS was the predominant cause of bloodstream infection

Overall, NTS (n = 896) accounted for 65.4% of confirmed BSI and 7.6% of suspected BSI sampled at Kisantu hospital. In children, NTS was the most frequently isolated pathogen in both children < 5 years and ≥ 5 years old (Fig 1) and represented 69.2% of all confirmed BSI. However, NTS BSI accounted for a significantly higher proportion of confirmed BSI in children < 5 years when compared to those ≥ 5 years (p<0.001). In adults, NTS represented 21.8% of all confirmed BSI and ranked second as the cause of BSI, following *Salmonella* Typhi (30.0% of all confirmed BSI). In Orientale, NTS (n = 37) were isolated from 3.2% of 1166 suspected BSI and from 28.2% of 131 confirmed BSI. In Equateur, NTS (n = 9) were isolated from 10.0% of 90 suspected BSI and in 50.0% of 18 confirmed BSI. NTS was the most frequent cause of BSI in both provinces. In Kinshasa, a pathogen was isolated from 102/525 (19.4%) suspected BSI, but no NTS were isolated during this surveillance period.

### Serotype distribution: O5-negative Salmonella Typhimurium has emerged

**Fig 2:**
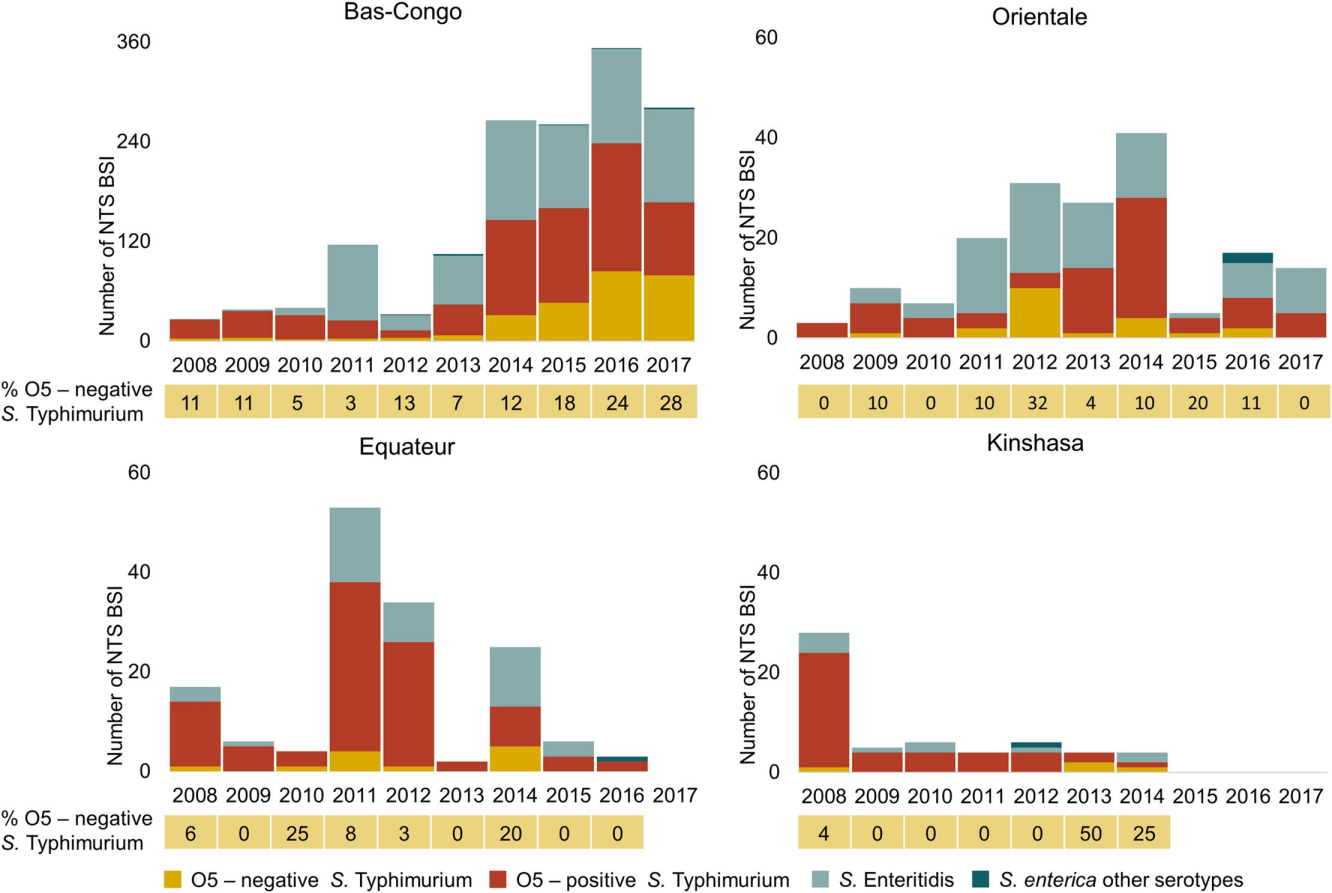
NTS-serotype distribution per year per province demonstrated variation of the predominant serotype over time and place and the emergence of O5-negative *Salmonella* Typhimurium. Bars represent the number of NTS bloodstream infection (BSI). Data below the bars represent the annual proportion (%) of O5-negative *Salmonella* typhimurium among the total NTS BSI.

At Kisantu hospital, *Salmonella* Typhimurium accounted for 63.1% (n = 566) of NTS BSI and *Salmonella* Enteritidis for 36.4% (n = 326). O5-negative *Salmonella* Typhimurium represented 36.9% of all *Salmonella* Typhimurium (O5+ (n = 357) and O5- (n = 209)) and 23.3% of all NTS BSI. The remaining 4 NTS isolates comprised 1 *Salmonella* Urbana, 1 *Salmonella* Chandans, 1 *Salmonella* Abony and 1 *Salmonella* serotype I:4,5:-:1,2. The serotype distribution did not significantly differ between children < 5 years, children ≥ 5 years and adults. The proportion of O5-negative *Salmonella* Typhimurium from all NTS increased yearly (Fig 2). In Orientale, O5-negative *Salmonella* Typhimurium only accounted for 8.1% (3/37) of NTS BSI. In Equateur, all isolated *Salmonella* Typhimurium (n = 5) were O5-positve.

### Patient characteristics: NTS mainly affected children under the age of two years

**Fig 3:**
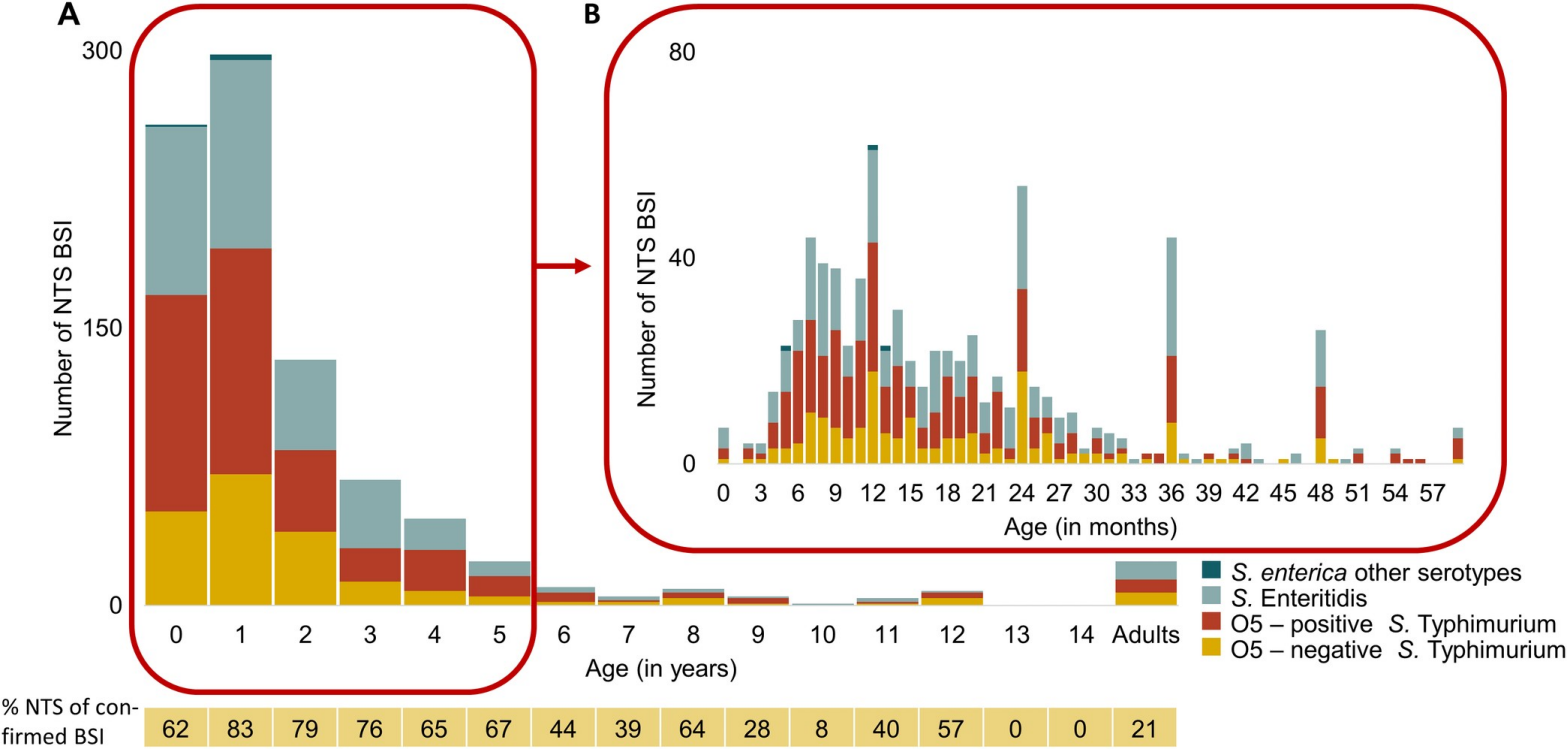
Age distribution of NTS bloodstream infections sampled at Kisantu general referral hospital from 2015–2017. **A:** Bars represent the number of NTS bloodstream infection (BSI) per age (in years). The proportion (%) of confirmed BSI episodes caused by NTS in the respective age group is displayed below the graph. **B:** Bars represent the number of NTS BSI per age (in months). During this period, 806/896 NTS BSI were obtained from children < 5 years old. The exact age in months was available for 770/806 NTS BSI from children < 5 years old. Poor knowledge and registration of the exact age in months resulted in artificial peaks of NTS on multiples of 12 months. Abbreviations: BSI: bloodstream infection.

At Kisantu general referral hospital, the median age of patients from which NTS were isolated was 17 months (P25-75: 10.5–30); 62.3% of NTS BSI occurred in children < 2 years and 89.9% in children < 5 years. NTS were isolated from 7 neonates (<28 days), from which 4 in the first week of life (on day 0-2-4-6). Accounting for only 2.7% (24/896) of NTS BSI, NTS BSI in adults was equally distributed over all ages. Fig 3 represents the age distribution of NTS and its serotypes. The median age of O5-positive *Salmonella* Typhimurium (15 months (P25-75: 10–26 months) was slightly lower compared to O5-negative *Salmonella* Typhimurium and *Salmonella* Enteritidis (18 months (P25-75: 12–30) and 19 months (P25-75: 11–36 months). Nevertheless, the age distributions of the serotypes were not significantly different (Wilcoxon-Mann-Whitney test: p = 0.06). Of all NTS, 55.8% was isolated from males (52.9% males for all suspected BSI) and this was similar for the different serotypes.

Four patients with recurrent BSI caused by NTS (1 *Salmonella* Enteritidis, 1 O5-positive *Salmonella* Typhimurium and 2 O5-negative *Salmonella* Typhimurium) were identified. The recurrences occurred 3–14 weeks after the initial episode in patients who were 1 to 5 years old.

### Antibiotic resistance profile: MDR was widespread and watch group antibiotics were affected

Data of antibiotic susceptibility testing were available for 864 out of 896 NTS isolates (96.4%) from Kisantu hospital. ***Table 2*** ogives an overview of the antibiotic resistance profiles per serotype. MDR was present in 87.4%, ceftriaxone resistance in 15.7%, azithromycin resistance in 14.9% and DCS in 7.3% of NTS isolates. Ceftriaxone resistance and azithromycin resistance co-occurred in 128/136 (94.1%) ceftriaxone resistant NTS isolates and in 128/129 (99.2%) azithromycin resistant NTS isolates. The median MIC of ciprofloxacin was 0.012 (IQR: 0.008–0.012) for all 3 main serotypes. Pefloxacin disk diffusion susceptibility testing detected all 63 DCS NTS without any false positive result. Nalidixic acid disk diffusion susceptibility testing failed to detect 2 DCS NTS, although diameters were borderline (19 and 20mm (breakpoint susceptibility ≥ 19mm [17])), there were no false positives. Prior use of antibiotics (<48 hours before blood culture sampling; data available for 11,757/11,764 (99.9%) suspected BSI) was observed in 24.0% of suspected BSI without pathogen isolation, in 26.6% of confirmed BSI and 24.9% of NTS BSI. Prior use of antibiotics was not significantly associated with MDR and DCS. However, prior use of antibiotics was associated with a higher proportion of azithromycin resistance (with prior antibiotic use: 19.2% of NTS vs. without: 13.5%, p = 0.04) and of ceftriaxone resistance (with prior antibiotic use: 19.6% of NTS vs. without: 14.5%, p = 0.07). Multidrug resistance was less frequent in adults (73.7% (14/19 NTS)) than in children (87.7% (741/845 NTS)), albeit not statistically significant (p = 0.07). The proportions of DCS, ceftriaxone or azithromycin resistance from all NTS isolates were not significantly different in children and adults. For all the tested antibiotics, proportions of resistant NTS isolates were similar in children < 5 years and ≥ 5 years old.

**Table 2:** Antibiotic resistance profile per NTS-serotype isolated at Kisantu general referral hospital from 2015–2017.

*% (N resistant isolates)*	*S*. Enteritidis (N = 316)	O5+ *S*. Typhimurium (N = 345)	O5- *S*. Typhimurium (N = 199)	All NTS (N = 864)
Ampicillin	86.1 (272)	92.5 (319)	97.5 (194)	91.1 (787)
Trimethoprim-sulfamethoxazole	84.2 (266)	95.1 (328)	97.5 (194)	91.4 (790)
Chloramphenicol	84.2 (266)	87.6 (302)	96.5 (192)	88.2 (762)
Multidrug resistant (MDR)	83.5 (264)	86.4 (298)	96.0 (191)	87.5 (755)
Ceftriaxone	0	38.0 (131)	2.0 (4)	15.7 (136)
Azithromycin	0	36.2 (125)	1.5 (3)	14.9 (129)
Decreased ciprofloxacin susceptibility (DCS)	2.2 (7)	13.3 (46)	4.5 (9)	7.3 (63)
**From which:**				
MDR + DCS	1.3 (4)	10.4 (36)	4.5 (9)	5.8 (50)
MDR + ceftriaxone + azithromycin (XDR)	0	34.8 (120)	1.0 (2)	14.2 (123)
MDR + ceftriaxone + azithromycin + DCS (PDR)	0	0.3 (1) *	0 *	0.1 (1)

Complete results of antibiotic susceptibility testing at reference level were available from 864/896 NTS isolates. The results of antibiotic susceptibility testing were not reported separately for other *Salmonella* serotypes (4/864). Data are presented in percentages with the corresponding number of NTS isolates between brackets.

In the other provinces, MDR was widespread as well (Orientale: 32/36 (88.9%) and Equateur 8/8 (100%) NTS isolates for which data on antibiotic susceptibility testing were available at reference level). Resistance to watch group antibiotics was only found in 1 NTS BSI. This isolate was sampled in Orientale and presented MDR, DCS, ceftriaxone and azithromycin resistance, *i*.*e*. PDR.

### Comparison with previous surveillance periods: NTS remain the primary cause of bloodstream infection in children

**Fig 4:**
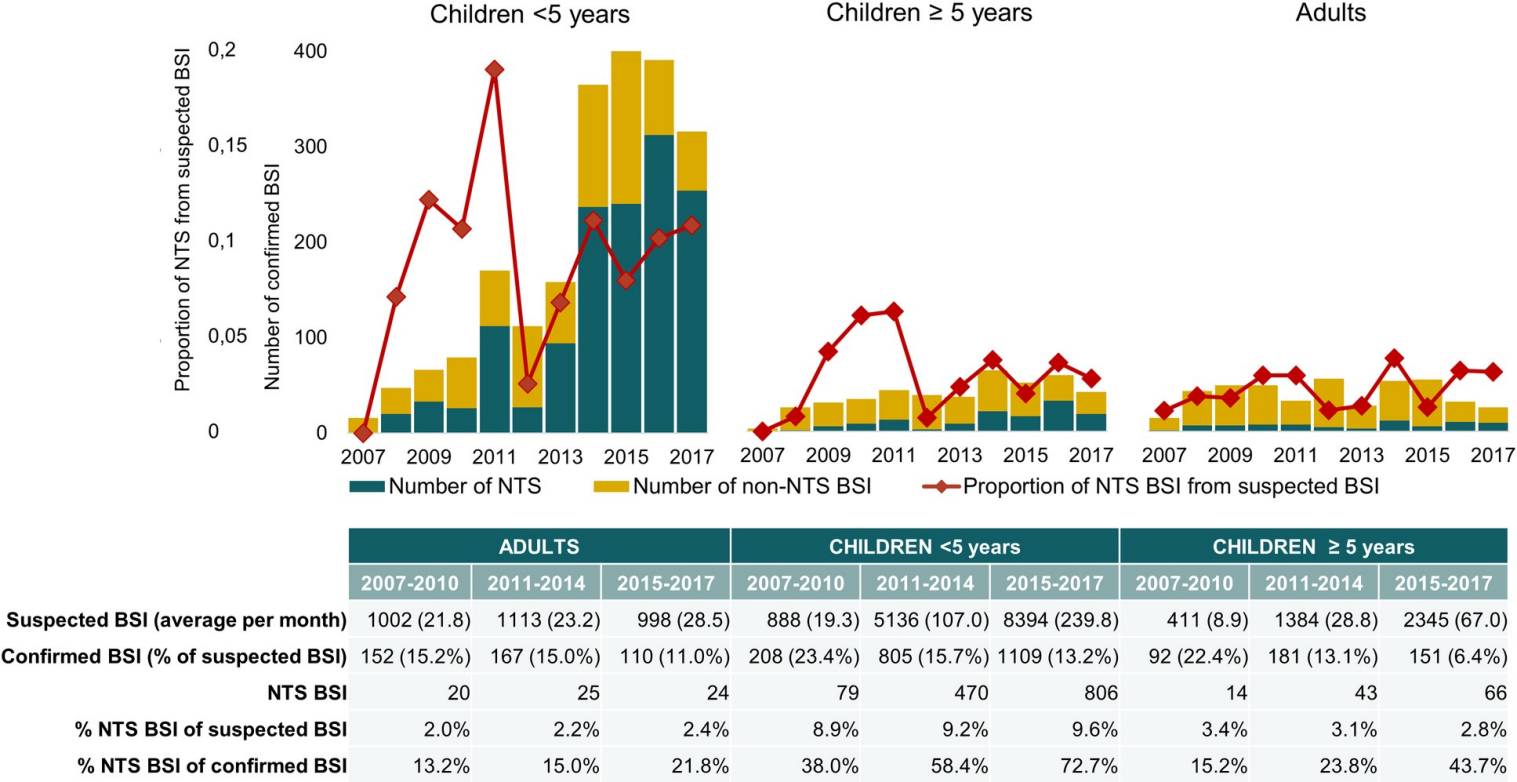
Longitudinal analysis of blood culture sampling and NTS isolation according to age group at Kisantu general referral hospital. Unless otherwise specified, data in the tables are displayed as the number of bloodstream infections (BSI). The longitudinal increase in the proportion of NTS BSI among all confirmed BSI in both children < 5 years and ≥ 5 years was statistically significant (p<0.001).

At Kisantu hospital, the number of NTS BSI increased longitudinally, particularly in children < 5 years (Fig 4). This increase was associated with an increase of the average number of suspected BSI per month. However, the proportion of NTS BSI from confirmed BSI increased significantly in children (p<0.001; Fig 4), whereas other pathogens, i.e. *Salmonella* Typhi, *Klebsiella* spp., *Staphylococcus* aureus and Gram-negative non-lactose fermenting bacteria, decreased. The latter was associated with a decrease in blood culture positivity rate, although it remained within the quality range. [25] The proportion of NTS BSI from all suspected BSI increased a little in children < 5 years, but this was not statistically significant (Fig 4).

### Comparison with previous surveillance periods: NTS-serotype distribution varies

As demonstrated in Fig 2, the predominant serotype varied over time at Kisantu hospital. In 2011, an outbreak of *Salmonella* Enteritidis occurred [32]. Since 2013, the proportion of O5-negative *Salmonella* Typhimurium from all NTS linearly increased with an average of 5.5% per year (p<0.001). Over all the surveillance periods, the Typhimurium–to–Enteritidis ratio varied between minimum 1 : 3.6 (2008) and maximum 1 : 0.04 (2008). In addition, the serotype distribution varied over the provinces. In 2011, an outbreak of O5-positive *Salmonella* Typhimurium occurred in Equateur. [33] In 2012, O5-negative *Salmonella* Typhimurium accounted for 10/13 (76.9%) *Salmonella* Typhimurium in Orientale. More detailed data on the spatiotemporal evolution of the serotype distribution can be found in S2 Table.

### Comparison with previous surveillance periods: resistance against watch group antibiotics increased

**Fig 5:**
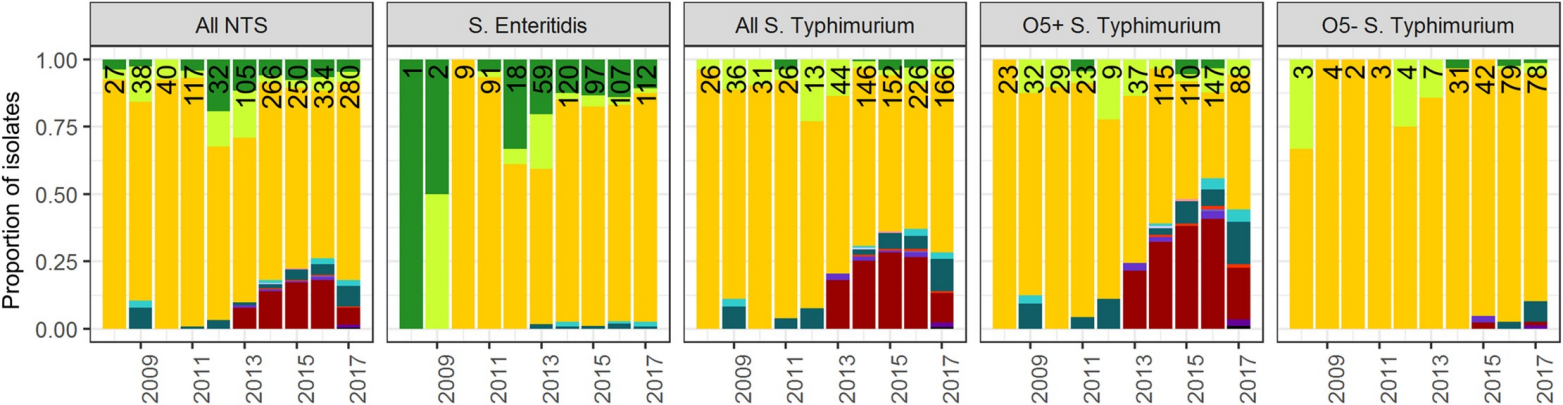
Proportional overview of antibiotic resistance in NTS bloodstream infections at Kisantu general referral hospital. Data on top of the bars represent the number of isolates per year. Overall, data from antibiotic susceptibility testing at reference level were available from 1490 NTS isolates, from which 253 were O5-negative *Salmonella* Typhimurium, 613 were O5-positive *Salmonella* Typhimurium, 617 were *Salmonella* Enteritidis and 7 were other *Salmonella* enterica serotypes. The latter were not displayed separately, due to their low number. The year 2007 was also not displayed, since only 1 NTS isolate was confirmed at Kisantu hospital during this year. Abbreviations: MDR: multidrug resistance, XDR: extensive drug resistance, PDR: pandrug resistance, Amp: ampicillin, SxT: trimethoprim-sulfamethoxazole, Clr: chloramphenicol, DCS: decreased ciprofloxacin susceptibility, CTRR: ceftriaxone resistance, AZIR: azithromycin resistance, *S*.: *Salmonella*, O5+: O5-antigen positive, O5-: O5-antigen negative.

**Fig 6:**
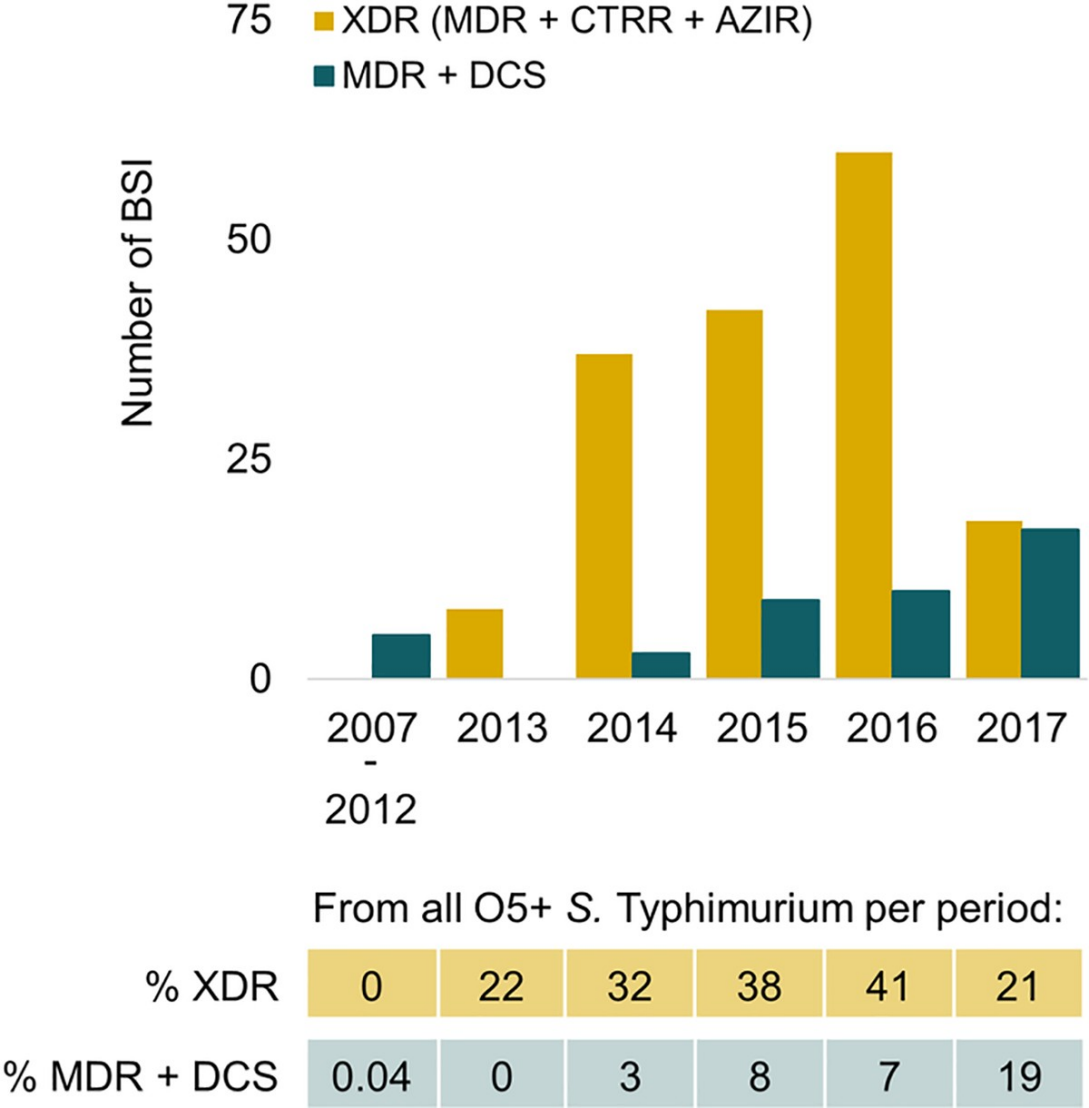
Longitudinal analysis of multidrug resistance combined with decreased ciprofloxacin susceptibility and extensive drug resistance in O5-positive *Salmonella* Typhimurium at Kisantu general referral hospital. Data below the graphs represent the proportion (%) of resistant isolates among all O5-positive *Salmonella* Typhimurium. Abbreviations: MDR: multidrug resistance, XDR: extensive drug resistance, DCS: decreased ciprofloxacin susceptibility, CTRR: ceftriaxone resistance, AZIR: azithromycin resistance, BSI: bloodstream infection, O5+ *S*. Typhimurium: O5-positive *Salmonella* Typhimurium.

At Kisantu hospital, the proportion of MDR was similar over the different surveillance periods, also when analyzed per age category. However, there was a significant increase in ceftriaxone resistance, azithromycin resistance and DCS (all p<0.005, Fig 5, more data can be found in S3 Table). Since 2013, XDR (MDR + ceftriaxone resistance + azithromycin resistance) and the combination of MDR with DCS emerged (Fig 6). Most XDR (165/168 (98.2%)) and MDR + DCS (44/60 (73.3%)) NTS were O5-positive *Salmonella* Typhimurium. The combination of MDR + DCS + ceftriaxone resistance, which is also an XDR-phenotype was found in 2 O5-positive *Salmonella* Typhimurium and 1 O5-negative *Salmonella* Typhimurium. In addition, XDR (MDR + ceftriaxone resistance + azithromycin resistance) in combination with DCS, *i*.*e*. PDR, was found in 1 O5-positive *Salmonella* Typhimurium isolated in 2017. In Kinshasa, a O5-positive Salmonella Typhimurium presenting PDR was found in 2008. In Orientale, an O5-negative *Salmonella* Typhimurium that presented PDR was isolated in 2016.

## Discussion

### Summary of findings

The present study confirmed the importance of NTS as the cause of more than half of confirmed BSI. In children < 5 years old, NTS were isolated from 9.6% of suspected BSI sampled at Kisantu general referral hospital between 2015–2017. This percentage slightly increased over the difference surveillance periods (2007–2017), albeit not statistically significant. The serotype distribution varied over time and province. At Kisantu general referral hospital, there was an annual increase of around 5.5% in the proportion of O5-negative *Salmonella* Typhimurium among NTS since 2013. The prevalence of NTS BSI was highest in children under the age of two years. MDR was widespread (83.5–96.0%) in all serotypes. In *Salmonella* Typhimurium, particularly in O5-positive ones, high proportions of XDR (MDR + ceftriaxone and azithromycin resistance; 34.8%) and MDR + DCS (10.4%) were found. Furthermore 3 *Salmonella* Typhimurium with PDR (MDR + ceftriaxone and azithromycin resistance + DCS) were observed over the successive surveillance periods, each originating from a different province.

### Limitations and strengths

This publication reports on the first decade of a national microbiological surveillance in DRC, a country where diagnostic microbiological facilities are nearly absent. Sampled and processed free of charge and embedded in the routine clinical practice of the participating health care facilities as a capacity building project, the microbiological surveillance network has built up the largest collection of NTS isolates in Central-Africa. Furthermore, the healthcare facility-based surveillance approach allowed to sample a large patient population, including the most vulnerable patients, in a cost-effective way. However, the healthcare facility-based approach also provoked some important limitations. Firstly, since no detailed data on population size, healthcare seeking behavior and referral itinerary were available, it was not possible to calculate population-based incidence rates. Secondly, the absence of a dedicated study team may have had an impact on the intensity and quality of blood culture sampling and laboratory work-up. The intensity and quality of blood culture surveillance were further challenged by the well-known technical, logistical an human resource constraints in a low-resource setting [34–36]. Thirdly, detailed clinical data were not registered as part of the surveillance. Hence, it is not possible to report on the clinical presentation, evolution and outcome of NTS BSI. Finally, the high level of antibiotic use prior to blood culture sampling impacts blood culture sensitivity and may have altered the distribution of pathogens isolated, e.g. the low number of Streptococc*us* pneumoniae.

### Comparison with other sub-Saharan African countries

Over the different surveillance periods, NTS consistently ranked first among bacteria causing BSI in children and the second in adults. The proportion of NTS among suspected BSI in children < 5 years old slightly increased, albeit not statistically significant. By contrast, the most recent calculations of the global NTS burden [37] were lower than previous estimates [3,38]. In Malawi [39], Tanzania [40], Kenya [41] and The Gambia [42], the incidence of NTS BSI decreased over time and was linked to declines in HIV-AIDS prevalence and *P*. *falciparum* malaria incidence. In DRC however, the already immense burden of *P*. *falciparum* is still increasing, which might explain the persistence of NTS causing BSI [10]. Alternatively, high disease transmission, high pathogenicity and altered health care seeking behavior might also play a role [4].

The serotype distribution varied each year and differed per province. Local outbreaks impacted the serotype distribution [32,33,43]. In line with previous reports from sub-Saharan African countries also consistently ranked *Salmonella* Typhimurium as the predominant serotype (representing 43–88% of NTS); the proportion of *Salmonella* Enteritidis (representing 10–50% of NTS) and other less frequent serotypes varied between countries [44]. Similar to the results presented in this study, the Typhimurium–to–Enteritidis ratio varied between 1:1 to 1:8 between countries [44]. Variations in serotype distribution over time or between centers have been recently described in Malawi, Burkina Faso and Mali [45–47].

This study is the first to report O5-negative *Salmonella* Typhimurium as an important cause of BSI. They were sporadically observed since 2008, but emerged gradually at Kisantu hospital since 2013. O5-negative *Salmonella* Typhimurium have been described as zoonotic pathogens (isolated from birds, cattle, swine and pets) that can cause foodborne illness in both industrialized and non-industrialized countries [48–55]. In accordance with its MDR-phenotype in the present study, the previously described zoonotic O5-negative *Salmonella* Typhimurium frequently carried various antibiotic resistance genes [48,50,54,56]. If the O5-negative *Salmonella* Typhimurium from the present study have a zoonotic origin, their emergence may reflect environmental changes influencing the animal reservoir. Alternatively, they could be descendants of *Salmonella* Typhimurium ST313, as genetic changes in the *oafA* gene can result in a loss of acetylation of the abequose residue of the O4 epitope and thus cause the loss of the O5 epitope [57,58]. This loss might have important consequences, as the O-antigen structure plays a role in pathogenicity and immunogenicity [59]. Of note, it is not excluded that O5-negative *Salmonella* Typhimurium account for some of the *Salmonella* Typhimurium BSI in other sub-Saharan African countries too, as the O5-antigen is not included in routine serotyping, particularly not in non-reference laboratories.

Over the years in DRC, MDR remained widespread in all serotypes, whereas recent data from Mali [47], Malawi [45,60], South-Africa [61], Kenya [62–64] and Ghana [65] showed MDR to be less frequent in *Salmonella* Enteritidis or more variable over time. Since 2013, resistance to ceftriaxone and azithromycin and DCS increased substantially in O5-positive *Salmonella* Typhimurium in DRC. For the period 2015–2017, a third of them was resistant to ceftriaxone and azithromycin and one out of eight were DCS. Pefloxacin disk diffusion methods flawlessly predicted DCS and nalidixic acid performed relatively well. A similar concurrent emergence of ceftriaxone resistance and DCS was seen in Kenya with 15% ceftriaxone resistance and 6% DCS in NTS (on a total of 281 NTS isolates) [66] and in South-Africa with ceftriaxone resistance in 9% of *Salmonella* Typhimurium (n = 2420) and DCS in around 30% of NTS (*Salmonella* Typhimurium: n = 2420; *Salmonella* Enteritidis: n = 1149), including some fully fluoroquinolone resistant isolates [61] In Mali, 18.2% of *Salmonella* Enteritidis were ceftriaxone resistant [47]. Recently, one DCS Salmonella Typhimurium was reported in Ghana [67] and NTS presenting ceftriaxone resistance (n = 4) or DCS (n = 1) were reported in Malawi [45]. Resistance to azithromycin in NTS BSI has not been reported in other sub-Saharan African countries yet, but this may be partially due to the absence of international consensus breakpoints to interpret azithromycin susceptibility testing [14,18]. The presence of a high proportion of O5-positive *Salmonella* Typhimurium presenting XDR and MDR + DCS is new and worrisome. Moreover, 3 PDR NTS isolates were found, leaving only carbapenems or other reserve antibiotics as possible, but in a low-resource setting mostly unaffordable, treatment options.

### Relevance and future research

It is probable that the persistence of NTS as the primary cause of BSI is linked to the high P.f. malaria incidence in DRC. However, to improve our understanding of the driving forces of this persistence, environmental and climate factors, including urbanization and climate change, should be studied. These factors can either directly influence NTS-disease transmission or indirectly impact NTS incidence *e*.*g*. by their effect on the incidence of *P*.f. malaria. A better understanding of the driving forces of NTS and ongoing efforts to elucidate the reservoir and transmission will direct public health interventions.

The persistence of NTS BSI and worrisome emergence of (co)resistance to key access and watch group antibiotics are a large public health threat. The potential benefits of a vaccine for NTS are clear, even more because of the probably human reservoir [4]. Several NTS vaccines are being developed and are progressing towards clinical trials [68]. Most NTS vaccines, *i*.*e*. the live attenuated candidates [69] (multivalent) glycoconjugate vaccines [70] and Generalized Modules for Membrane Antigens based vaccines [71], are O-antigen based. As this report shows rapid changes in serotype distribution, multivalent vaccines are needed. The risk for serotype replacement after vaccine introduction, as known from streptococci [72] and meningococci [73], is real and vigilant monitoring of NTS-serotypes is warranted. Furthermore, the absence of the O5-epitope can impact vaccine immunogenicity of this strain. Alongside with *in vitro* and *in vivo* vaccine efficacy studies, the burden and genomics of O5-negative *Salmonella* Typhimurium should be clarified.

In an era of limited treatment options, antibiotic stewardship becomes even more important. After the early emergence of widespread MDR in NTS [1,4,5], antibiotic treatment has often relied on watch group antibiotics. Treatment of NTS with ceftriaxone or ciprofloxacin is experience-based [5], i.e. to the best of our knowledge, no clinical study has assessed the treatment efficacy of any of the different antibiotic treatment options. In line with *Salmonella* Typhi, azithromycin is a possible alternative for oral treatment in case of DCS, although validated breakpoints to interpret azithromycin susceptibility testing are still pending. The favorable pharmacokinetic profile of azithromycin with good oral absorption, intracellular accumulation and long half-life could make it the ideal candidate for intravenous to oral switch of antibiotic treatment [74,75]. Its value in the initial treatment of NTS BSI can be questioned as high plasma concentrations are warranted in sepsis [76]. Full treatment with azithromycin monotherapy is efficacious in typhoid fever, but typhoidal serovars do not typically elicit septic shock [77]. Dedicated observational studies and clinical trials assessing the pharmacokinetics, pharmacodynamics and treatment efficacy of watch group antibiotics are urgently needed to develop evidence-based treatment guidelines for NTS BSI.

### Conclusion

This present study reports valuable information for surveillance, treatment and disease prevention (public health interventions and vaccination) of NTS BSI. Blood culture surveillance in DRC from 2015–2017 confirmed NTS as the major cause of BSI, particularly in young children. In contrast to other countries, the frequency of NTS BSI did not decline over the years, which might possibly be explained by the increasing incidence of *P*. *falciparum* malaria in DRC. In Bas-Congo, O5-negative *Salmonella* Typhimurium are emerging since 2013 and increased up to 28.1% of all NTS BSI in 2017. As the absence of the O5-epitope can impact immunogenicity and therefore vaccine development, the O5 antigen should actively be monitored in reference settings. Antibiotic resistance to watch group antibiotics was observed in a substantial proportion of NTS isolates. Moreover, combined resistance, incl. XDR and PDR-phenotypes has emerged in O5-positive *Salmonella* Typhimurium. Field studies assessing the efficacity of different antibiotic treatment regimens are urgently needed to feed evidence-based antibiotic stewardship.

## Supporting information

S1 STROBE Checklist Checklist of items that should be included in reports of observational studies. (DOC)

S1 Table Democratic Republic of the Congo country profile according to reference 1–4. (DOCX)

S2 Table Annual distribution of NTS-serotypes compiled for Democratic Republic of the Congo and per province. Both the number of BSI per serotype and their respective proportion of all NTS BSI (percentage in italic) are shown.
(DOCX)

S3 Table Longitudinal analysis of the antibiotic susceptibility profile of NTS isolates from Kisantu general referral hospital for which data from antibiotic susceptibility testing at reference level were available (n = 1490). Data are presented in percentages with the corresponding number of NTS isolates between brackets for the most recent and total surveillance period. * For 2007–2010: results from cefotaxime instead of ceftriaxone susceptibility testing ** For 2015–2017: From which 3 MDR + DCS + ceftriaxone R isolate.
(DOCX)
