# Supplementary material for: Non-typhoidal Salmonella bloodstream infections in Kisantu, DR Congo: Emergence of O5-negative Salmonella Typhimurium and extensive drug resistance
Source: PLoS Negl Trop Dis. 2020 Apr 2;14(4):e0008121. doi: 10.1371/journal.pntd.0008121 (PMC7156106; doi:10.1371/journal.pntd.0008121)
Supplement: S1 STROBE Checklist — (DOC) [file pntd.0008121.s001.doc]

**STROBE Statement — checklist of items that should be included in reports of observational studies**

|  | Item No | Recommendation | Section & paragraph |  |
| --- | --- | --- | --- | --- |
| **Title and abstract** | 1 | (*a*) Indicate the study’s design with a commonly used term in the title or the abstract | Abstract – methodology paragraph |  |
|  |  | (*b*) Provide in the abstract an informative and balanced summary of what was done and what was found | Abstract – methodology, results & conclusions paragraph |  |
| Introduction | | | |  |
| Background/rationale | 2 | Explain the scientific background and rationale for the investigation being reported | Introduction: whole section |  |
| Objectives | 3 | State specific objectives, including any prespecified hypotheses | Introduction: last paragraph |  |
| Methods | | | |  |
| Study design | 4 | Present key elements of study design early in the paper | Methodology – Study setting & period: first paragraph |  |
| Setting | 5 | Describe the setting, locations, and relevant dates, including periods of recruitment, exposure, follow-up, and data collection | Methodology – Study setting & period: all paragraphs |  |
| Participants | 6 | Give the eligibility criteria, and the sources and methods of selection of participants | Methodology - Blood culture indications, sampling and culturing: first paragraph |  |
| Variables | 7 | Clearly define all outcomes, exposures, predictors, potential confounders, and effect modifiers. Give diagnostic criteria, if applicable | Methodology – Reference testing: serotyping and antibiotic susceptibility testing: last paragraph & Definitions and terminology & Comparison with previous surveillance periods |  |
| Data sources/ measurement | 8* | For each variable of interest, give sources of data and details of methods of assessment (measurement). Describe comparability of assessment methods if there is more than one group | Methodology - Blood culture indications, sampling and culturing & Reference testing: serotyping and antibiotic susceptibility testing & Comparison with previous surveillance periods |  |
| Bias | 9 | Describe any efforts to address potential sources of bias | Methodology - Comparison with previous surveillance periods |  |
| Study size | 10 | Explain how the study size was arrived at | NA (report of blood culture surveillance) |  |
| Quantitative variables | 11 | Explain how quantitative variables were handled in the analyses. If applicable, describe which groupings were chosen and why | Methodology – Blood culture indications, sampling and culturing: first three lines of second paragraph & Reference testing: serotyping and antibiotic susceptibility testing: last paragraph |  |
| Statistical methods | 12 | (*a*) Describe all statistical methods, including those used to control for confounding | Methodology – Statistical analysis |  |
|  |  | (*b*) Describe any methods used to examine subgroups and interactions | Methodology – Statistical analysis |  |
|  |  | (*c*) Explain how missing data were addressed | Methodology – Statistical analysis  Results - Patient characteristics: NTS mainly affects children under the age of two years: first two lines  Results - Antibiotic resistance profile: MDR is widespread and watch group antibiotics are in danger: first two lines |  |
|  |  | (*d*) If applicable, describe analytical methods taking account of sampling strategy | NA |  |
|  |  | Describe any sensitivity analyses | NA |  |
| Results | | | | |
| Participants | 13* | (a) Report numbers of individuals at each stage of study—eg numbers potentially eligible, examined for eligibility, confirmed eligible, included in the study, completing follow-up, and analysed | Results – Whole section | |
|  |  | (b) Give reasons for non-participation at each stage | Results - Patient characteristics: NTS mainly affects children under the age of two years: first two lines  Results - Antibiotic resistance profile: MDR is widespread and watch group antibiotics are in danger: first two lines | |
|  |  | (c) Consider use of a flow diagram | Considered for overview of sampling, but already published in Tack et al, Clinical Infectious Diseases 2019. We preferred to refer to this publication to avoid duplication. | |
| Descriptive data | 14* | (a) Give characteristics of study participants (eg demographic, clinical, social) and information on exposures and potential confounders | Results - Patient characteristics: NTS mainly affects children under the age of two years: first two lines | |
|  |  | (b) Indicate number of participants with missing data for each variable of interest | Results - Patient characteristics: NTS mainly affects children under the age of two years: first two lines | |
| Outcome data | 15* | Report numbers of outcome events or summary measures | Results – whole section | |
| Main results | 16 | (*a*) Give unadjusted estimates and, if applicable, confounder-adjusted estimates and their precision (eg, 95% confidence interval). Make clear which confounders were adjusted for and why they were included | Results – whole section | |
|  |  | (*b*) Report category boundaries when continuous variables were categorized | Methodology - Blood culture indications, sampling and culturing & Reference testing: serotyping and antibiotic susceptibility testing & Comparison with previous surveillance periods | |
|  |  | (*c*) If relevant, consider translating estimates of relative risk into absolute risk for a meaningful time period | NA | |
| Other analyses | 17 | Report other analyses done—eg analyses of subgroups and interactions, and sensitivity analyses | NA | |
| Discussion | | | | |
| Key results | 18 | Summarise key results with reference to study objectives | Discussion – summary of findings | |
| Limitations | 19 | Discuss limitations of the study, taking into account sources of potential bias or imprecision. Discuss both direction and magnitude of any potential bias | Discussion – limitations & strengths | |
| Interpretation | 20 | Give a cautious overall interpretation of results considering objectives, limitations, multiplicity of analyses, results from similar studies, and other relevant evidence | Discussion - comparison with other sub-Saharan African countries & relevance and future research  Conclusion | |
| Generalisability | 21 | Discuss the generalisability (external validity) of the study results | Discussion - comparison with other sub-Saharan African countries | |
| Other information | | | | |
| Funding | 22 | Give the source of funding and the role of the funders for the present study and, if applicable, for the original study on which the present article is based | Stated during online application at PLOS Neglected Tropical Diseases | |

*Give information separately for cases and controls in case-control studies and, if applicable, for exposed and unexposed groups in cohort and cross-sectional studies.

**Note:** An Explanation and Elaboration article discusses each checklist item and gives methodological background and published examples of transparent reporting. The STROBE checklist is best used in conjunction with this article (freely available on the Web sites of PLoS Medicine at http://www.plosmedicine.org/, Annals of Internal Medicine at http://www.annals.org/, and Epidemiology at http://www.epidem.com/). Information on the STROBE Initiative is available at www.strobe-statement.org.
