# Supplementary material for: Non-typhoidal Salmonella bloodstream infections in Kisantu, DR Congo: Emergence of O5-negative Salmonella Typhimurium and extensive drug resistance
Source: PLoS Negl Trop Dis. 2020 Apr 2;14(4):e0008121. doi: 10.1371/journal.pntd.0008121 (PMC7156106; doi:10.1371/journal.pntd.0008121)
Supplement: S1 Table — (DOCX) [file pntd.0008121.s002.docx]

## Supporting table S1:

**Supporting Table S1.** Democratic Republic of the Congo country profile according to reference 1-4.

| Area | 2.340.000 km^2^ |
| --- | --- |
| Paved roads | 2794 km |
| Population | 77.267.000 |
| under 15 years | 45% |
| median age | 17 years |
| life expectancy at birth | 59.1 years |
| Population with access to improved |  |
| water source | 52% |
| sanitation facilities | 29% |
| Gross national income per capita, PPP | 680 int. $ |
| total expenditure on health | 4% of GDP |
| Human development index | 0.435 |
| world rank (number of total countries) | 176/188 |
| expected/mean years of schooling | 9.8/6.1 |
| adult literacy rate (>15 years) | 77.3% |
| population living below income poverty line, $1.9/day | 77.1% |
| Health |  |
| under-five mortality rate (of life births) | 98.3/1000 |
| moderate or severe stunting (< 5 years) | 42.6% |
| deaths due to malaria | 106.6/100.000 |
| share of estimated cases of malaria of Central Africa | 59% |
| HIV prevalence (15-49 years) | 0.8% |

Abbreviations: PPP, purchasing power parity; GDP, gross domestic product; HIV, human immunodeficiency virus.

**References**

1. The World Factbook. Democratic republic of the Congo. 2018. <https://www.cia.gov/library/publications/the-world-factbook/geos/cg.html>
2. World Health Organization. Democratic Republic of the Congo: WHO statistical profile. 2015. <http://www.who.int/countries/cod/en/>
3. The World Bank. Data: Dem. Rep. Congo. 2017. <http://www.worldbank.org/en/country/drc>
4. United Nations Development Programme. Human development Report: Congo (Democratic Republic of the). 2016. <http://hdr.undp.org/en/countries/profiles/COD>
