# Supplementary material for: Non-typhoidal Salmonella bloodstream infections in Kisantu, DR Congo: Emergence of O5-negative Salmonella Typhimurium and extensive drug resistance
Source: PLoS Negl Trop Dis. 2020 Apr 2;14(4):e0008121. doi: 10.1371/journal.pntd.0008121 (PMC7156106; doi:10.1371/journal.pntd.0008121)
Supplement: S2 Table — Both the number of BSI per serotype and their respective proportion of all NTS BSI (percentage in italic) are shown. (DOCX) [file pntd.0008121.s003.docx]

## Supporting table S2:

| **Democratic Republic of the Congo** | **2007** | **2008** | **2009** | **2010** | **2011** | **2012** | **2013** | **2014** | **2015** | **2016** | **2017** | **2007-2017** |
| --- | --- | --- | --- | --- | --- | --- | --- | --- | --- | --- | --- | --- |
| O5+ *Salmonella* Typhimurium | 14 | 63 | 47 | 40 | 63 | 41 | 54 | 147 | 120 | 162 | 94 | 845 |
|  | *60,9%* | *81,8%* | *79,7%* | *70,2%* | *32,5%* | *39,8%* | *39,1%* | *43,9%* | *44,1%* | *43,2%* | *31,8%* | *43,8%* |
| O5- *Salmonella* Typhimurium | 0 | 5 | 5 | 3 | 9 | 15 | 10 | 41 | 47 | 86 | 79 | 300 |
|  | *0,0%* | *6,5%* | *8,5%* | *5,3%* | *4,6%* | *14,6%* | *7,2%* | *12,2%* | *17,3%* | *22,9%* | *26,7%* | *15,6%* |
| *Salmonella* Enteritidis | 8 | 9 | 7 | 14 | 121 | 45 | 72 | 147 | 104 | 121 | 121 | 769 |
|  | *34,8%* | *11,7%* | *11,9%* | *24,6%* | *62,4%* | *43,7%* | *52,2%* | *43,9%* | *38,2%* | *32,3%* | *40,9%* | *39,9%* |
| *Salmonella* enterica other serotypes | 1 | 0 | 0 | 0 | 0 | 2 | 2 | 0 | 1 | 4 | 2 | 12 |
|  | *4,3%* | *0,0%* | *0,0%* | *0,0%* | *0,0%* | *1,9%* | *1,4%* | *0,0%* | *0,4%* | *1,1%* | *0,7%* | *0,6%* |
| All NTS | 23 | 77 | 59 | 57 | 194 | 103 | 138 | 335 | 272 | 375 | 296 | 1929 |

**Supporting table S2**. Annual distribution of NTS-serotypes compiled for Democratic Republic of the Congo and per province

| **Kinshasa** | **2007** | **2008** | **2009** | **2010** | **2011** | **2012** | **2013** | **2014** | **2015** | **2016** | **2017** | **2007-2017** |
| --- | --- | --- | --- | --- | --- | --- | --- | --- | --- | --- | --- | --- |
| O5+ *Salmonella* Typhimurium | 14 | 23 | 4 | 4 | 4 | 4 | 2 | 1 | 0 | 0 | 0 | 56 |
|  | *63,6%* | *82,1%* | *80,0%* | *66,7%* | *100,0%* | *66,7%* | *50,0%* | *25,0%* |  |  |  | *70,9%* |
| O5- *Salmonella* Typhimurium | 0 | 1 | 0 | 0 | 0 | 0 | 2 | 1 | 0 | 0 | 0 | 4 |
|  | *0,0%* | *3,6%* | *0,0%* | *0,0%* | *0,0%* | *0,0%* | *50,0%* | *25,0%* |  |  |  | *5,1%* |
| *Salmonella* Enteritidis | 7 | 4 | 1 | 2 | 0 | 1 | 0 | 2 | 0 | 0 | 0 | 17 |
|  | *31,8%* | *14,3%* | *20,0%* | *33,3%* | *0,0%* | *16,7%* | *0,0%* | *50,0%* |  |  |  | *21,5%* |
| *Salmonella* enterica other serotypes | 1 | 0 | 0 | 0 | 0 | 1 | 0 | 0 | 0 | 0 | 0 | 2 |
|  | *4,5%* | *0,0%* | *0,0%* | *0,0%* | *0,0%* | *16,7%* | *0,0%* | *0,0%* |  |  |  | *2,5%* |
| All NTS | 22 | 28 | 5 | 6 | 4 | 6 | 4 | 4 | 0 | 0 | 0 | 79 |

| **Bas-Congo** | **2007** | **2008** | **2009** | **2010** | **2011** | **2012** | **2013** | **2014** | **2015** | **2016** | **2017** | **2007-2017** |
| --- | --- | --- | --- | --- | --- | --- | --- | --- | --- | --- | --- | --- |
| O5+ Salmonella Typhimurium | 0 | 23 | 32 | 29 | 22 | 9 | 37 | 115 | 114 | 154 | 88 | 623 |
|  | *0,0%* | *85,2%* | *84,2%* | *72,5%* | *18,8%* | *28,1%* | *35,2%* | *43,4%* | *43,7%* | *43,5%* | *31,3%* | *41,0%* |
| O5- Salmonella Typhimurium | 0 | 3 | 4 | 2 | 3 | 4 | 7 | 31 | 46 | 84 | 79 | 263 |
|  | *0,0%* | *11,1%* | *10,5%* | *5,0%* | *2,6%* | *12,5%* | *6,7%* | *11,7%* | *17,6%* | *23,7%* | *28,1%* | *17,3%* |
| Salmonella Enteritidis | 1 | 1 | 2 | 9 | 91 | 18 | 59 | 120 | 100 | 114 | 112 | 627 |
|  | *100,0%* | *3,7%* | *5,3%* | *22,5%* | *77,8%* | *56,3%* | *56,2%* | *45,3%* | *38,3%* | *32,2%* | *39,9%* | *41,2%* |
| Salmonella enterica other serotypes | 0 | 0 | 0 | 0 | 0 | 1 | 2 | 0 | 1 | 1 | 2 | 7 |
|  | *0,0%* | *0,0%* | *0,0%* | *0,0%* | *0,0%* | *3,1%* | *1,9%* | *0,0%* | *0,4%* | *0,3%* | *0,7%* | *0,5%* |
| All NTS | 1 | 27 | 38 | 40 | 117 | 32 | 105 | 265 | 261 | 354 | 281 | 1521 |

| **Equateur** | **2007** | **2008** | **2009** | **2010** | **2011** | **2012** | **2013** | **2014** | **2015** | **2016** | **2017** | **2007-2017** |
| --- | --- | --- | --- | --- | --- | --- | --- | --- | --- | --- | --- | --- |
| O5+ *Salmonella* Typhimurium | 0 | 13 | 5 | 3 | 34 | 25 | 2 | 8 | 3 | 2 | 0 | 95 |
|  |  | *76,5%* | *83,3%* | *75,0%* | *64,2%* | *73,5%* | *100,0%* | *32,0%* | *50,0%* | *66,7%* |  | *63,3%* |
| O5- *Salmonella* Typhimurium | 0 | 1 | 0 | 1 | 4 | 1 | 0 | 5 | 0 | 0 | 0 | 12 |
|  |  | *5,9%* | *0,0%* | *25,0%* | *7,5%* | *2,9%* | *0,0%* | *20,0%* | *0,0%* | *0,0%* |  | *8,0%* |
| *Salmonella* Enteritidis | 0 | 3 | 1 | 0 | 15 | 8 | 0 | 12 | 3 | 0 |  | 42 |
|  |  | *17,6%* | *16,7%* | *0,0%* | *28,3%* | *23,5%* | *0,0%* | *48,0%* | *50,0%* | *0,0%* |  | *28,0%* |
| *Salmonella* enterica other serotypes | 0 | 0 | 0 | 0 | 0 | 0 | 0 | 0 | 0 | 1 | 0 | 1 |
|  |  | *0,0%* | *0,0%* | *0,0%* | *0,0%* | *0,0%* | *0,0%* | *0,0%* | *0,0%* | *33,3%* |  | *0,7%* |
| All NTS | 0 | 17 | 6 | 4 | 53 | 34 | 2 | 25 | 6 | 3 | 0 | 150 |

| **Orientale** | **2007** | **2008** | **2009** | **2010** | **2011** | **2012** | **2013** | **2014** | **2015** | **2016** | **2017** | **2007-2017** |
| --- | --- | --- | --- | --- | --- | --- | --- | --- | --- | --- | --- | --- |
| O5+ *Salmonella* Typhimurium | 0 | 3 | 6 | 4 | 3 | 3 | 13 | 24 | 3 | 6 | 5 | 70 |
|  |  | *100,0%* | *60,0%* | *57,1%* | *15,0%* | *9,7%* | *48,1%* | *58,5%* | *60,0%* | *33,3%* | *35,7%* | *39,8%* |
| O5- *Salmonella* Typhimurium | 0 | 0 | 1 | 0 | 2 | 10 | 1 | 4 | 1 | 2 | 0 | 21 |
|  |  | *0,0%* | *10,0%* | *0,0%* | *10,0%* | *32,3%* | *3,7%* | *9,8%* | *20,0%* | *11,1%* | *0,0%* | *11,9%* |
| *Salmonella* Enteritidis | 0 | 0 | 3 | 3 | 15 | 18 | 13 | 13 | 1 | 7 | 9 | 82 |
|  |  | *0,0%* | *30,0%* | *42,9%* | *75,0%* | *58,1%* | *48,1%* | *31,7%* | *20,0%* | *38,9%* | *64,3%* | *46,6%* |
| *Salmonella* enterica other serotypes | 0 | 0 | 0 | 0 | 0 | 0 | 0 | 0 | 0 | 2 | 0 | 2 |
|  |  | *0,0%* | *0,0%* | *0,0%* | *0,0%* | *0,0%* | *0,0%* | *0,0%* | *0,0%* | *11,1%* | *0,0%* | *1,1%* |
| All NTS | 0 | 3 | 10 | 7 | 20 | 31 | 27 | 41 | 5 | 18 | 14 | 176 |

Legend: Both the number of BSI per serotype and their respective proportion of all NTS BSI (percentage in italic) are shown.
