# Supplementary material for: Non-typhoidal Salmonella bloodstream infections in Kisantu, DR Congo: Emergence of O5-negative Salmonella Typhimurium and extensive drug resistance
Source: PLoS Negl Trop Dis. 2020 Apr 2;14(4):e0008121. doi: 10.1371/journal.pntd.0008121 (PMC7156106; doi:10.1371/journal.pntd.0008121)
Supplement: S3 Table — Data are presented in percentages with the corresponding number of NTS isolates between brackets for the most recent and total surveillance period. * For 2007–2010: results from cefotaxime instead of ceftriaxone susceptibility testing ** For 2015–2017: From which 3 MDR + DCS + ceftriaxone R isolate. (DOCX) [file pntd.0008121.s004.docx]

## Supporting table S3:

**Supporting table S3**. Longitudinal analysis of the antibiotic susceptibility profile of NTS isolates from Kisantu general referral hospital for which data from antibiotic susceptibility testing at reference level were available (n = 1490)

| **Resistant isolates:** | **2015-2017 (N = 864)** | **2011 - 2014 (n = 520)** | **2007 - 2010 (n = 106)** | **2007-2017 (n = 1490)** |
| --- | --- | --- | --- | --- |
| Ampicillin | 91.1 (787) | 90.6 | 96.2 | 91.3 (1360) |
| Trimethoprim-sulfamethoxazole | 91.4 (790) | 88.5 | 95.3 | 90.7 (1351) |
| Chloramphenicol | 88.2 (762) | 85.6 | 90.6 | 87.5 (1303) |
| Multidrug resistant (MDR) | 87.4 (755) | 84.4 | 87.7 | 86.4 (1287) |
| Ceftriaxone* | 15.7 (136) | 9.5 | 0 | 12.4 (185) |
| Decreased ciprofloxacin susceptibility (DCS) | 7.3 (63) | 1.9 | 3.8 | 5.2 (77) |
| Azithromycin | 14.9 (129) | 9.4 | 0 | 12.0 (178) |
| **From which:** |  |  |  |  |
| MDR + DCS ** | 5.8 (50) | 1.3 | 2.7 | 4.0 (60) |
| MDR + ceftriaxone + azithromycin | 14.2 (123) | 8.7 | 0 | 11.3 (168) |
| MDR + ceftriaxone + azithromycin + DCS | 0.1 (1) | 0 | 0 | 0.1 (1) |
| *Legend: Data are presented in percentages with the corresponding number of NTS isolates between brackets for the most recent and total surveillance period.* | | | | |
| ** For 2007-2010: results from cefotaxime instead of ceftriaxone susceptibility testing* | | |  |  |
| *** For 2015-2017: From which 3 MDR + DCS + ceftriaxone R isolate* | |  |  |  |
